# *Phalaenopsis* orchid miniaturization by overexpression of *OsGA2ox6,* a rice GA2-oxidase gene

**DOI:** 10.1186/s40529-020-00288-0

**Published:** 2020-04-06

**Authors:** Kun-Ting Hsieh, Su-Hui Liu, I-Wen Wang, Liang-Jwu Chen

**Affiliations:** 1 Institute of Molecular Biology, National Chung Hsing University, Taichung, 40227 Taiwan; 2 Division of Biotechnology, Taiwan Agriculture Research Institute, Taichung, 41362 Taiwan; 3 Agricultural Biotechnology Center, National Chung Hsing University, Taichung, 40227 Taiwan

**Keywords:** *Phalaenopsis* orchids, Miniature flowers, Transgenic, GA2-oxidase, *OsGA2ox6*

## Abstract

**Background:**

Phalaenopsis orchids are one of the most common potted orchids sold worldwide. Most Phalaenopsis cultivars have long inflorescences that cause shipping problems and increase handling costs. Miniaturization of Phalaenopsis orchids not only reduces overall production costs but also can expand the appeal of the orchids to a different group of consumers who prefer to keep flowers on desks or tabletops. Although some miniature Phalaenopsis plants can be obtained via hybridization or mutation, they are unpredictable and limited in variety. We therefore used the transgenic approach of overexpressing gibberellin 2-oxidase 6 (*OsGA2ox6*), a rice GA deactivation gene, to investigate its functional effect in miniaturizing Phalaenopsis and to create a stable miniaturization platform to facilitate a supply for the potential demands of the miniature flower market.

**Results:**

A commercial moth orchid, *Phalaenopsis* Sogo Yukidian ‘SPM313’, was transformed with the plasmid vector *Ubi:OsGA2ox6* and successfully overexpressed the *OsGA2ox6* gene *in planta*. The transgenic lines displayed darker-green, shorter, and wider leaves, thicker roots and much shorter flower spikes (10 cm vs 33 cm) than the *nontransgenic* line with a normal flower size and blooming ability and are therefore an ideal miniaturized form of Phalaenopsis orchids.

**Conclusions:**

We demonstrated that the ectopic expression of *OsGA2ox6* can miniaturize *Phalaenopsis* Sogo Yukidian ‘SPM313’ while preserving its blooming ability, providing an alternative, useful method for miniaturizing Phalaenopsis species. This miniaturization by a transgenic approach can be further expanded by using GA2ox genes from different plant species or different gene variants, thereby expanding the technical platform for miniaturizing Phalaenopsis species to meet the potential demands of the miniature Phalaenopsis flower market.

## Background

Orchidaceae is one of the largest families of flowering plants and contains some of the most important ornamental plants sold on the market. From a floriculture standpoint, orchids have great economic value; the total number of commercially traded live orchid plants in the global market increased from 460 million in 1996–2005 to 660 million in 2006–2015, an increase of approximately 200 million plants (44%) (Hinsley et al. 2017). The orchid family comprises 763 genera and more than 28 thousand species (Christenhusz and Byng 2016; Govaerts et al. 2015). Among them, species of the genus *Phalaenopsis*, also known as moth orchids, are some of the most commonly sold potted orchids worldwide (Hinsley et al. 2017). Although the *Phalaenopsis* genus has great commercial value, most Phalaenopsis cultivars have large flowers and long inflorescences/flower spikes; these bulky and cumbersome features cause shipping problems and increase handling costs.

For commercial production in floriculture markets, plant growth retardants (PGRs), a group of synthetic bioregulators, have been widely used to shape plant morphology, e.g., to create miniature Phalaenopsis orchids (Lopez et al. 2007). For instance, the PGRs paclobutrazol (PAC) and uniconazole (UNI) are frequently used to suppress internode elongation in floriculture crops by inhibiting gibberellin (GA) biosynthesis at the enzyme ent-kaurene oxidase (KO) (Additional file 1: Fig S1) (Rademacher 2000). The suppression of inflorescence elongation by PAC has also been demonstrated in Phalaenopsis cultivars (Newton and Runkle 2010; Wang and Hsu 1994). However, repetitive PAC treatment is needed for long-term inhibition of inflorescence growth, and several factors, such as PAC concentration, treatment temperature, timing and application method, all influence the effectiveness of PAC treatment (Barrett and Bartuska 1982; Gilbertz 1992; Latimer and Whipker 2013), which increases the complexity of PGR application in the floriculture industry.

Miniature Phalaenopsis orchids not only benefit commercial producers by reducing handling costs but also may be desired by a different group of consumers. Miniature orchids are easier to keep in small pots and are much more convenient to display indoors or on a tabletop if large floor spaces are not available. Even though miniature orchids such as *Phalaenopsis equestris* have been used as breeding parents to reduce plant stature (Frowine 2007; Hus and Chen 2015), introducing miniature orchids into a breeding program often produces progeny with small flowers that are not desirable in the market.

In addition to PGR treatments and selection via hybridization or mutation, genetic engineering is an alternative approach for shaping plant morphology. Many miniaturized transgenic plants have been produced by manipulating GA biosynthesis through the ectopic expression of GA deactivation genes (Lo et al. 2017; Nir et al. 2014; Wuddineh et al. 2015). GA deactivation enzymes such as GA methyltransferase, elongated uppermost internode (EUI) and GA2-oxidase (GA2ox) have been identified, and plant height reduction by the overexpression of these genes has been demonstrated (Lo et al. 2008; Sakamoto et al. 2001; Varbanova et al. 2007; Zhu et al. 2006). Therefore, overexpressing these GA deactivation genes ectopically in Phalaenopsis orchids through a transgenic approach that can miniaturize the orchids is logically feasible. However, appropriate miniaturized transgenic plants with normal flowers are rare, as many aspects of floral development are impaired by severe dwarfism (Hedden, 2003; Lee et al. 2014; Lo et al. 2008; Sakamoto et al. 2001, 2003; Singh et al. 2002); this complication has hindered the use of these GA deactivation genes in the flower industry.

Although GA deactivation can be achieved through various modifications (Yamaguchi 2008), 2-beta hydroxylation by GA2oxs seems to be the most conserved GA deactivation process among plant species (Gou et al. 2011; Huang et al. 2015; Kim et al. 2019; Lo et al. 2008; Schomburg et al. 2003; Schrager-Lavelle et al. 2019; Wuddineh et al. 2015). GA2oxs can be further classified into the C_19_ and C_20_ types, which are responsible for catalyzing C_19_-GAs and C_20_-GAs, respectively. Although ectopic expression of both types of GA2oxs can miniaturize plants, in comparison to the expression of C_19_-type GA2ox, that of C_20_-type GA2oxs has little effect on flower and seed development (Lo et al. 2008; Sakamoto et al. 2001; Schomburg et al. 2003). Our previous study showed that the expression of the C_20_-type GA2ox *OsGA2ox6* had no effect on the reproductive organs of rice and tobacco (Lo et al. 2008). This characteristic makes *OsGA2ox6* a suitable gene for miniaturizing Phalaenopsis orchids.

In this study, transgenic Phalaenopsis orchids overexpressing *OsGA2ox6* were generated. We examined the effects of ectopic OsGA2ox6 on development and discussed the potential application of these transgenic plants.

## Materials and methods

### Plant materials, propagation of PLBs and growth conditions

Protocorm-like bodies (PLBs) of the commercialized *Phalaenopsis* variety Sogo Yukidian ‘SPM313’ were purchased from Sunhope Biotech (Chiayi, Taiwan). When the PLBs grew to approximately 2–4 mm, their tip portions were cut horizontally (Additional file 1: Fig S2) to generate additional PLBs. The cut PLBs were cultured in T2 medium containing 2% (w/v) sweet potato homogenate, 2.5% (w/v) banana pulp, 0.35% (w/v) Hyponex No. 1 (Hyponex Co. Ltd., Japan), 0.1% (w/v) tryptone, 0.01% (w/v) citric acid, 2% (w/v) sucrose, 0.1% (w/v) charcoal, and 0.3% (w/v) phytagel at pH 5.5 (Chan et al. 2005) for propagation, and the newly propagated PLBs were used for transformation. Petri dishes (90 × 15 mm) and glass vases (80 × 120 mm) were used for PLB propagation and plantlet regeneration after agrobacterium-mediated transformation. Individual 2.5- or 3-inch pots filled with Chilean sphagnum moss (Chile) were used to grow the regenerated plantlets, which were first grown in a growth chamber at 28/23 °C under a 16 h/8 h light–dark cycle for approximately 2 months and then moved into a greenhouse under natural environmental conditions until blooming.

### Construction of the *Ubi:OsGA2ox6* transformation vector

The full-length cDNA of *OsGA2ox6* (1077 bp) was amplified with an *OsGA2ox6*-*BamHI*-*F & R* primer set (Additional file 2: Table S1) by Phusion High-Fidelity DNA Polymerase (Thermo Fisher Scientific) and cloned (by *BamHI* digestion) into pAHC18 plasmid, which is a modified version of pUC18 that contains the maize *Ubiquitin* (*Ubi*) promoter and *Nopaline Synthase* (*Nos*) terminator (Bruce et al. 1989). The *OsGA2ox6*-containing pAHC18 was further inserted into the binary vector *pCAM1301* (Hajdukiewicz et al. 1994) to obtain the transformation vector *Ubi:OsGA2ox6*. The resulting plasmid vector in *Agrobacterium tumefaciens* strain EHA-105 was used for Phalaenopsis transformation.

### Phalaenopsis transformation and plantlet regeneration

When the new PLBs grew to approximately 2–4 mm, their tip portions were cut horizontally (Additional file 1: Fig S2) and cultured in T2 medium 7 days before transformation. The transformation vector *Ubi:OsGA2ox6* in *Agrobacterium tumefaciens* strain EHA-105 was cultured in AB medium (bioWORLD, Ohio) at 28 °C 2 days before transformation. The Phalaenopsis transformation was based on methods reported previously (Chan et al. 2005). On the day of transformation, the cut PLBs were incubated with acetosyringone (AS)-induced *Ubi:OsGA2ox6*-containing *Agrobacterium tumefaciens* EHA-105 for 30 min, and then the infected PLBs were moved to Petri dishes containing T2 medium plus AS (200 μM) and incubated in the dark at 25 °C for 3 days before selection. During selection, the PLBs were washed several times with MS medium containing 500 μg/mL cefotaxime (Cyrusbioscience, Seattle) to cleanse the *Agrobacterium* and then placed in T2 medium containing 500 μg/mL cefotaxime plus 5 μg/mL hygromycin at 25 °C under a 16 h/8 h light–dark cycle for 30 days as the first round of selection. The PLBs surviving after selection were further dissected and subcultured monthly on T2 medium plus 25 μg/mL hygromycin for transgenic plantlet regeneration. Transgenic plantlets were maintained by either dissecting the regenerated plantlets or mass propagation through horizontally cut transgenic PLBs.

### PCR, RT-PCR and southern blot analyses

Using CTAB buffer extraction protocols (Doyle and Doyle 1987), the genomic DNA (~ 0.1 µg) extracted from the leaf tissue of *Phalaenopsis* Sogo Yukidian ‘SPM313’, the nontransgenic (*NT)* line and the *OsGA2ox6*-overexpression (*GA2ox6*-*OX)* transgenic lines were subjected to PCR analysis with gene-specific primer sets (Additional file 2: Table S1) and DreamTaq DNA polymerase (Thermo Fisher Scientific) for transgene identification.

For RT-PCR analysis, total RNA was extracted from the leaf tissue using TRIzol reagent (Invitrogen) and then treated with RNase-free DNase I (Thermo Fisher Scientific) to remove any possible DNA contaminants. Three micrograms of the DNA-free RNA sample was used as a template for cDNA synthesis (reverse transcription) with a RevertAid first-strand cDNA synthesis kit (Thermo Fisher Scientific) using the protocols recommended by the manufacturer in a 20 µl total reaction volume. Then, 1 µl of the synthesized cDNA was subjected to PCR analysis in a 15 μl total reaction volume using a gene-specific primer set (Additional file 2: Table S1) and GoTaq DNA polymerase (Promega) for transgene expression analysis.

For Southern blot analysis, 30 µg of each DNA sample was digested by HindIII, subjected to 1% (w/v) agarose gel analysis and then transferred to Amersham Hybond-N^+^ membrane (GE Healthcare) according to the manufacturer’s instructions. The P^32^-labeled GUS DNA probe was used for hybridization according to the instructions for the Amersham Rediprime II DNA labeling system (GE Healthcare).

### GUS activity assay

A GUS activity assay was performed as described previously (Hsing et al. 2007), with minor modifications. Approximately 5 mm of leaf or root tissue was excised from the plantlet and placed in GUS staining solution, and vacuum aspiration was applied for 30 min. Then, the samples were incubated overnight at 37 °C. The staining solution and chlorophyll were removed by incubation with 70% ethanol at 65 °C for 1 h. The GUS staining solution contained 80 mM sodium phosphate buffer (pH 7.0), 0.4 mM potassium ferricyanide (pH 7.0), 8 mM EDTA, 0.05% (w/v) Triton X-100, and 2 mM X-glucuronide.

## Results

### Generation of *OsGA2ox6*-overexpressing (*GA2ox6*-*OX*) transgenic Phalaenopsis orchids

**Fig. 1:**
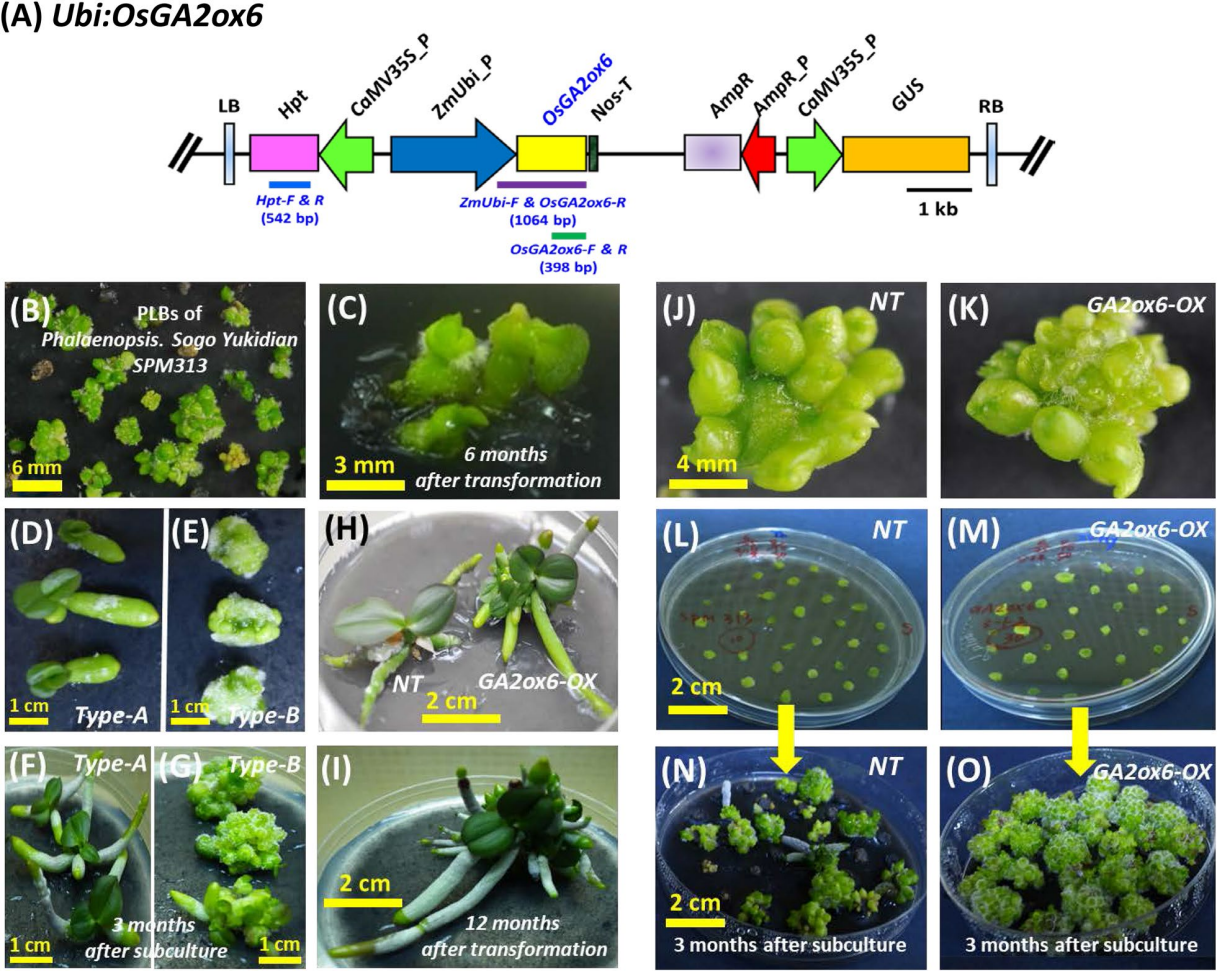
*Ubi:OsGA2ox6* transformation vector and comparisons of different propagation types of *GA2ox6*-*OX* transgenic lines at various growth stages. **a** Schematic diagram of the *Ubi:OsGA2ox6* transformation vector. The target gene *OsGA2ox6* (yellow box) driven by the *ZmUbi* promoter (blue arrow), hygromycin phosphotransferase (*Hpt*) and GUS genes driven by the 35S promoter (green arrows) in their relative scale within the left border (LB) and right border (RB) of T-DNA are shown. The primers (*Hpt*-*F &* -*R* and *ZmUbi*-*F & OsGA2ox6*-*R*) used for confirming vector integration (genomic DNA PCR) and the primers (*OsGA2ox6*-*F &* -*R*) used for gene expression analysis (RT-PCR) as well as the sizes in bp of their PCR products are indicated. **b** Protocorm-like bodies (PLBs) of *Phalaenopsis* Sogo Yukidian *‘*SPM313’ used for transformation. **c** Three surviving transgenic PLBs 6 months after transformation and selection. **d–g** Growth of propagation type A (**d**, **f**) and type B (**e**, **g**) from the *GA2ox6*-*OX* transgenic line 1 month (**d**, **e**) and 3 months (F, G) after subculture. A group of three PLBs is shown. **h** Examples of an *NT* (left) and a *GA2ox6*-*OX* transgenic (right) plantlet growing multiple leaves and roots approximately 10 months after transformation. **i** Close-up view of a transgenic plantlet growing approximately 12 months after transformation. **j**–**k** Example of the mother PLBs from *NT* (**j**) and *GA2ox6*-*OX* transgenic (**k**) lines used for propagation. **l**–**m** Clonal propagation of *NT* and *GA2ox6*-*OX* transgenic PLBs compared in parallel. The PLBs from *NT* (**l**) and *GA2ox6*-*OX* (**m**) lines were dissected horizontally to expose their surrounding epidermal cells in order to generate and propagate new PLBs. **n**–**o** Growth of many PLBs three months after subculture for *NT* (**n**) and *GA2ox6*-*OX* (**o**) lines

To investigate how a rice C_20_-type GA2oxs can affect the growth of Phalaenopsis orchid plants and be used as a method to miniaturize Phalaenopsis species for the floriculture market, a plasmid (*Ubi:OsGA2ox6*) (Fig. 1a) with the *OsGA2ox6* gene driven by a maize ubiquitin promoter was introduced into PLBs of *Phalaenopsis* Sogo Yukidian *‘*SPM313’ (Fig. 1b) by agrobacterium-mediated transformation. Six months after transformation, three surviving PLB groups that survived out of approximately four hundred PLBs on the selection medium (medium containing 25 μg/mL hygromycin) started to grow new PLBs, and the leaf-like organs regenerated (Fig. 1c). The surviving PLBs were dissected and subcultured in a selection medium on a monthly basis, and two types of propagation patterns were observed (Fig. 1d, e) in all *GA2ox6*-*OX* transgenic lines. Type A grew roots and leaves normally (Fig. 1d). Type B grew more PLBs mostly from dissected/wounded regions (Fig. 1e). Three months after subculturing, type A grew more roots and leaflets (Fig. 1f), while type B grew large numbers of PLBs with fewer differentiated roots and leaflets (Fig. 1g). Close-up views of the *NT* line showed longer leaves (left side in Fig. 1h), and the *GA2ox6*-*OX* transgenic line showed shorter and wider leaves and thicker roots (right side in Fig. 1h). Approximately 12 months after transformation, a *GA2ox6*-*OX* transgenic line through type A propagation pattern grew multiple leaves and roots (Fig. 1i).

### PLBs of the *GA2ox6*-*OX* transgenic lines propagate much more quickly than those of the *NT* line and generate more PLBs from wounded regions

In the regeneration and propagation process, all the *GA2ox6*-*OX* transgenic lines revealed different proliferation and differentiation patterns (Fig. 1d, e) from those of *NT* plants. During the subculturing process, we observed that all the *GA2ox6*-*OX* transgenic lines generated PLBs efficiently from the dissection/wound sites, so their PLBs propagated much faster than those of the *NT* line. To explore whether this phenomenon can be maintained in subsequent subculture processes, the PLBs from the *NT* and *GA2ox6*-*OX* transgenic lines (Fig. 1j, k) were cut horizontally (Additional file 1: Fig S2) to expose their surrounding epidermal cells to generate new PLBs (Fig. 1l, m). Three months after subculture, the PLBs of the *GA2ox6*-*OX* transgenic lines propagated much faster and produced many more PLBs than the *NT* plants (Fig. 1n, o), with fewer differentiated plantlets.

### Molecular characterization of *GA2ox6*-*OX* transgenic plants

**Fig. 2:**
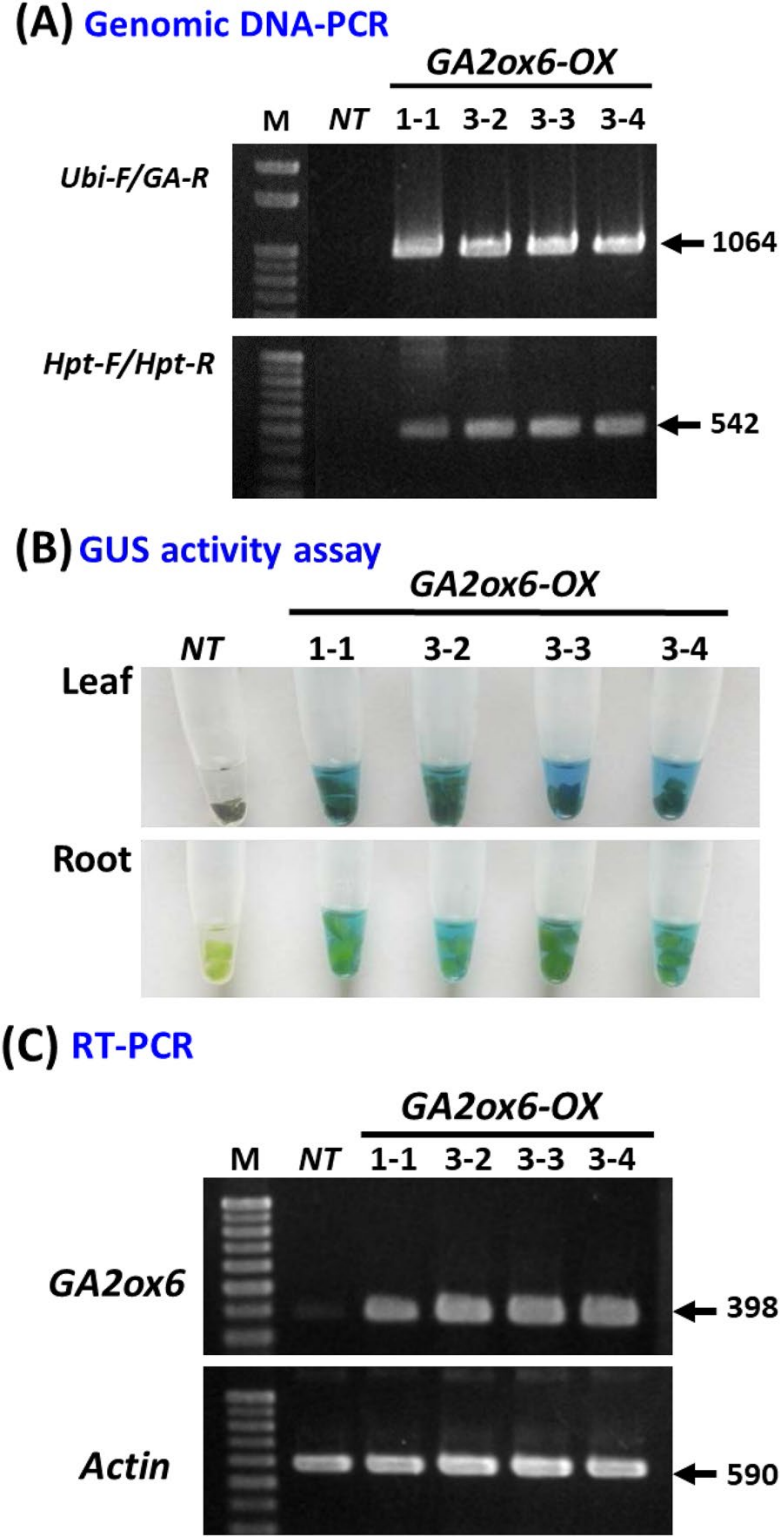
Molecular characterization of *GA2ox6*-*OX* transgenic lines. **a** Genomic DNA PCR. Primers specific to the T-DNA vector construct (*Ubi*-*F/GA*-*R*) and the *Hpt* gene (*Hpt*-*F/Hpt*-*R*) were used to verify the integration of the transgene. Four *GA2ox6*-*OX* transgenic lines, (#1-1, #3-2, #3-3 and #3-4), were analyzed. *NT*: nontransgenic line. **b** GUS activity assay. Leaf and root samples from the *NT* line and four *GA2ox6*-*OX* lines were analyzed. **c** RT-PCR. The *OsGA2ox6* transgene expression in the *NT* line and four *GA2ox6*-*OX* lines was analyzed by RT-PCR, and the expression of the actin gene (from Phalaenopsis) was used as an internal control

During the study period, three surviving PLB groups were originally obtained: PLB group #1 had only one PLB that survived and regenerated and was named #1-1; PLB group #2 had no survival; and PLB group #3 had 3 separate PLBs that survived and regenerated (as shown in Fig. 1c) and were named lines #3-2, #3-3 and #3-4. The regenerated plantlets (T_0_ plants) from these four putative *GA2ox6*-*OX* transgenic lines were analyzed by PCR using a primer set specific to a hygromycin B phosphotransferase (*Hpt*) gene (Fig. 1a, with 542 bp expected) and another primer set targeting the maize ubiquitin promoter and the 3’ end of *OsGA2ox6* (Fig. 1a, with 1064 bp expected) in the T-DNA construct to verify transgene integration. The results showed positive and correct PCR products for all four tested lines (Fig. 2a), confirming that they were *GA2ox6*-*OX* transgenic lines. In addition, both the leaf and root samples from these lines displayed a deep-blue color with X-Gluc substrate in the GUS activity assay (Fig. 2b), indicating that the *GUS* gene in the pCAMBIA1301 vector was also successfully integrated into the rice genome and expressed. Representative GUS staining results for PLBs and root tips of *NT* and *GA2ox6*-*OX* transgenic lines are provided as supplementary data in Additional file 1: Fig S3. The expression of the *OsGA2ox6* transgene measured by RT-PCR analyses showed a high level in all four transgenic lines but not in the *NT* line (Fig. 2c), indicating that the *OsGA2ox6* transgene was strongly expressed in the *GA2ox6*-*OX* transgenic lines. Furthermore, the leaf DNA samples from three (lines #1-1, #3-2, and #3-3) of the four *GA2ox6*-*OX* transgenic lines were analyzed by Southern blotting to verify their T-DNA insertion events (Additional file 1:Fig S4). The results of the different hybridization patterns suggested that each line was independently transformed and regenerated.

### Effects of ectopic OsGA2ox6 on vegetative development

**Fig. 3:**
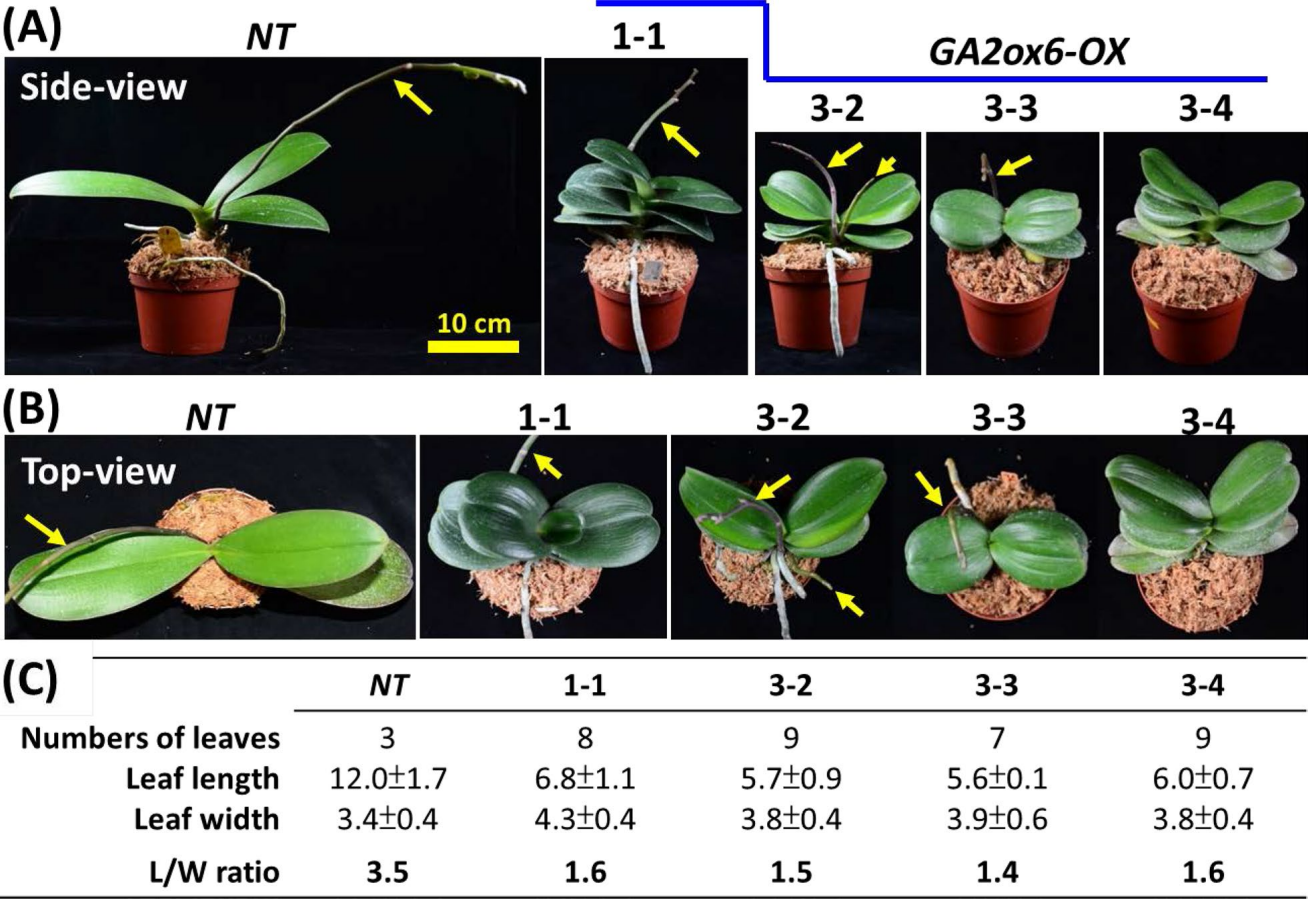
Phenotypic comparisons of the *NT* and *GA2ox6*-*OX* transgenic lines at the vegetative stage. Comparison of representative mature *NT* plants and four different *GA2ox6*-*OX* transgenic lines (#1-1, #3-2, #3-3 and #3-4) in side view (**a**) and top view (**b**). The numbers of leaves and the average leaf length, width and length/width ratio of each representative plant were measured for the *NT* and *GA2ox6*-*OX* transgenic lines (**c**), and the resulting data are shown below the pictures. The flower spikes are indicated by yellow arrows in the photos

**Fig. 4:**
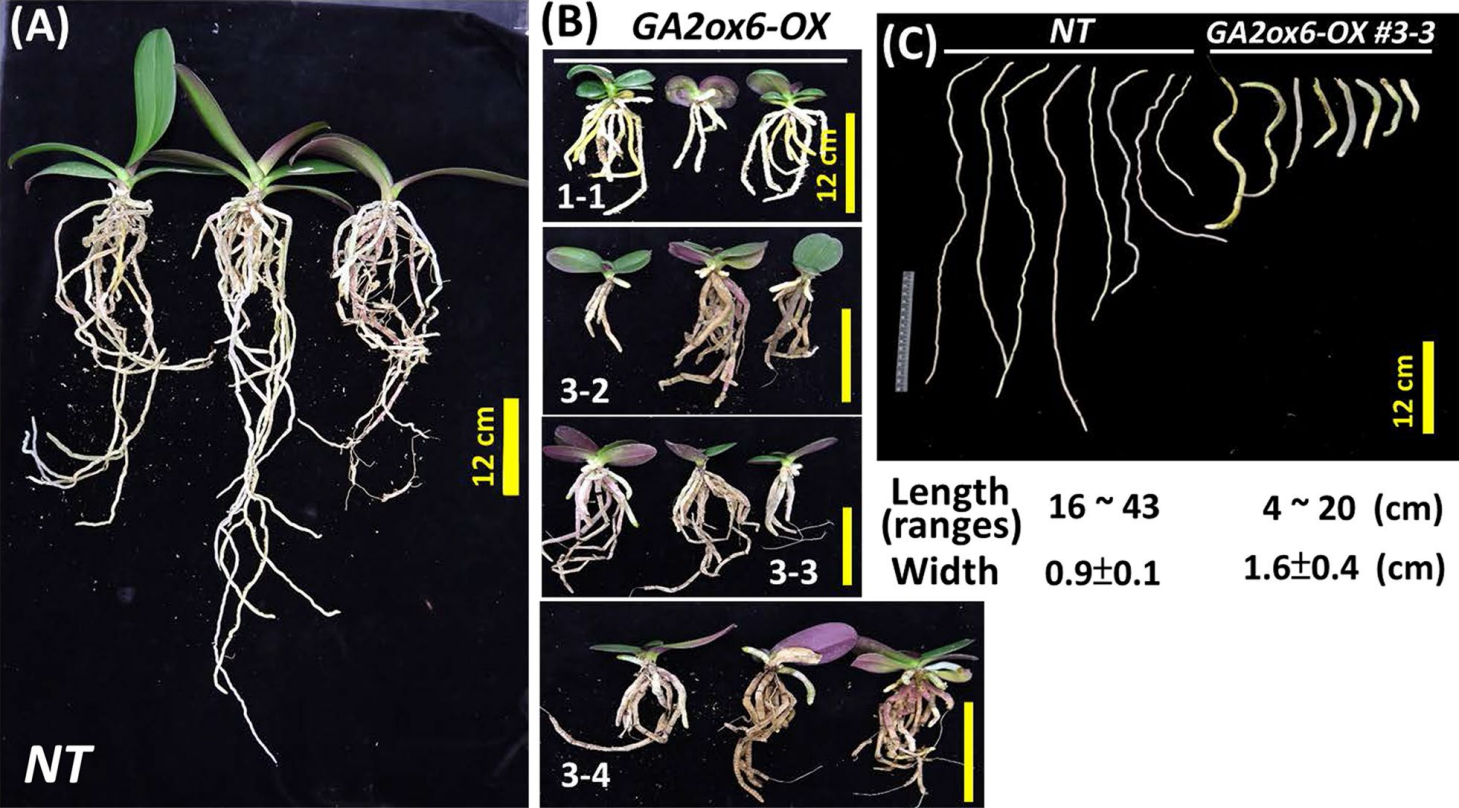
Phenotypic comparisons of roots of the *NT* and *GA2ox6*-*OX* transgenic lines. **a** Three plantlets of *NT* control plants showing longer roots. **b** Three plantlets from each of the four *GA2ox6*-*OX* transgenic lines (#1-1, #3-2, #3-3, and #3-4) showing shorter roots. Bar = 12 cm. **c** Comparison of the lengths and widths of roots dissected from the *NT* line and representative *GA2ox6*-*OX* transgenic line #3-3 are shown. The ranges of root length and average root width measured for the dissected roots are provided below the pictures

The regenerated transgenic plantlets were continuously subcultured and propagated to produce more orchid plants. After approximately 2 years of growing in a greenhouse under natural environmental conditions (with an average summer temperature of ~ 32/26 °C and average winter temperature of ~ 24/16 ^°^C), the transgenic orchid plants became mature and started to grow flower spikes (Fig. 3). The side view (Fig. 3a), top view (Fig. 3b) and leaf lengths, widths and numbers (Fig. 3c) for each representative plant of the four *GA2ox6*-*OX* transgenic lines and their *NT* host plants are shown for comparison and characterization (Fig. 3). All leaves, except the youngest one in line #1-1, were measured at the growth stage shown in Fig. 3a, b. The leaf shape and root sizes of *GA2ox6*-*OX* transgenic lines were very different from those of their *NT* host plants (Figs. 3, 4). The *GA2ox6*-*OX* transgenic lines displayed darker-green leaves with shorter lengths (5.6 to 6.8 cm in Fig. 3c), approximately half those in the *NT* line (12.0 cm in Fig. 3c), and greater widths (3.8–4.3 cm) than the average width in the *NT* line (3.4 cm), making the L/W ratio ~ 1.5 for the *GA2ox6*-*OX* transgenic lines and 3.5 for the *NT* line (Fig. 3c). In addition, three plants each from the *NT* line and four *GA2ox6*-*OX* transgenic lines (the growth stage of plants from line #1-1 was earlier than that of plants from lines #3-2, #3-3 and #3-4) were compared, and all *GA2ox6*-*OX* transgenic lines revealed similar phenotypes but showed different phenotypes compared with those of the *NT* line (Additional file 1: Fig S5). The length and width of the longest leaf were measured for three plants each of the *NT* line and transgenic lines #1-1, #3-2, #3-3, and #3-4 and showed the same tendency as those in Fig. 3c, although some variations were observed (Table 1). For example, *GA2ox6*-*OX* lines #3-2, #3-3 and #3-4 showed similar L/W ratios, while line #1-1 (at an earlier stage) had a lower L/W ratio (0.9) compared to those shown in Fig. 3c (1.6). The *GA2ox6*-*OX* lines produced 7-9 leaves, while only 3 leaves were produced in the *NT* line under the same growth conditions (Fig. 3c).

**Table 1:** Morphological comparison of leaves and roots in nontransgenic (*NT*) and *GA2ox6-OX* transgenic lines

	NT	1-1^a^	3-2	3-3	3-4
Leaf length^b^ (cm)	17.6 ± 3.2	5.3 ± 1.0^**^	6.8 ± 0.5^**^	5.8 ± 0.5^**^	7.9 ± 0.3^**^
Leaf width^c^ (cm)	6.2 ± 0.9	6.0 ± 0.6	5.2 ± 0.5	5.5 ± 0.4	5.8 ± 0.3
Leaf L/W ratio^d^	2.9 ± 0.9	0.9 ± 0.1^**^	1.3 ± 0.2^**^	1.1 ± 0.1^**^	1.4 ± 0.1^**^
Root length^e^ (cm)	54.9 ± 16.6	14.3 ± 4.3**	13.3 ± 8.8^**^	19.3 ± 11.6^**^	21.8 ± 1.6^**^
Root width^f^ (cm)	1.8 ± 0.1	2.1 ± 0.2**	2.8 ± 0.4^**^	3.1 ± 0.4^**^	2.4 ± 0.3^**^

Three plants each from the *NT* line and four *GA2ox6-OX* transgenic lines (#1-1, #3-2, #3-3 and #3-4) were measured and compared

^a^ Plants of line #1-1 were grown in 9 cm pots at a relative early stage (Additional file 1: Fig S5). Others were grown in 10.5 cm pots

^b^ Leaf length average ± SE of the longest leaf from 3 individual plants

^c^ Leaf width average ± SE of the same longest leaf from the above 3 individual plants

^d^ Ratio of leaf length to leaf width

^e^ Root length average ± SE of the longest root from 3 individual plants

^f^ Root width average ± SE of the thickest root from 3 individual plants

** Average ± SE (n = 3) showed a significant difference from the *NT* control with a p-value < 0.01

Compared to those of the *NT* line (Fig. 4a), the roots of the *GA2ox6*-*OX* transgenic lines (Fig. 4b, lines #1-1, #3-2, #3-3 and #3-4) were much shorter and thicker. The measurements from a representative plant line #3-3 showed that the root length ranged from 4 to 20 cm and was much smaller than the 16 to 43 cm root length of the *NT* line (Fig. 4c); the average root width (1.6 cm) in the *GA2ox6*-*OX* transgenic line was much greater than that (0.9 cm) in the *NT* line (Fig. 4c). Moreover, the greatest root length and root width values collected from three plants each of the *NT* line and four transgenic lines (#1-1, #3-2, #3-3 and #3-4) revealed the same pattern, with a much shorter root length and thicker root width compared to those of the *NT* line (Table 1).

### Effects of ectopic OsGA2ox6 on reproductive development

**Fig. 5:**
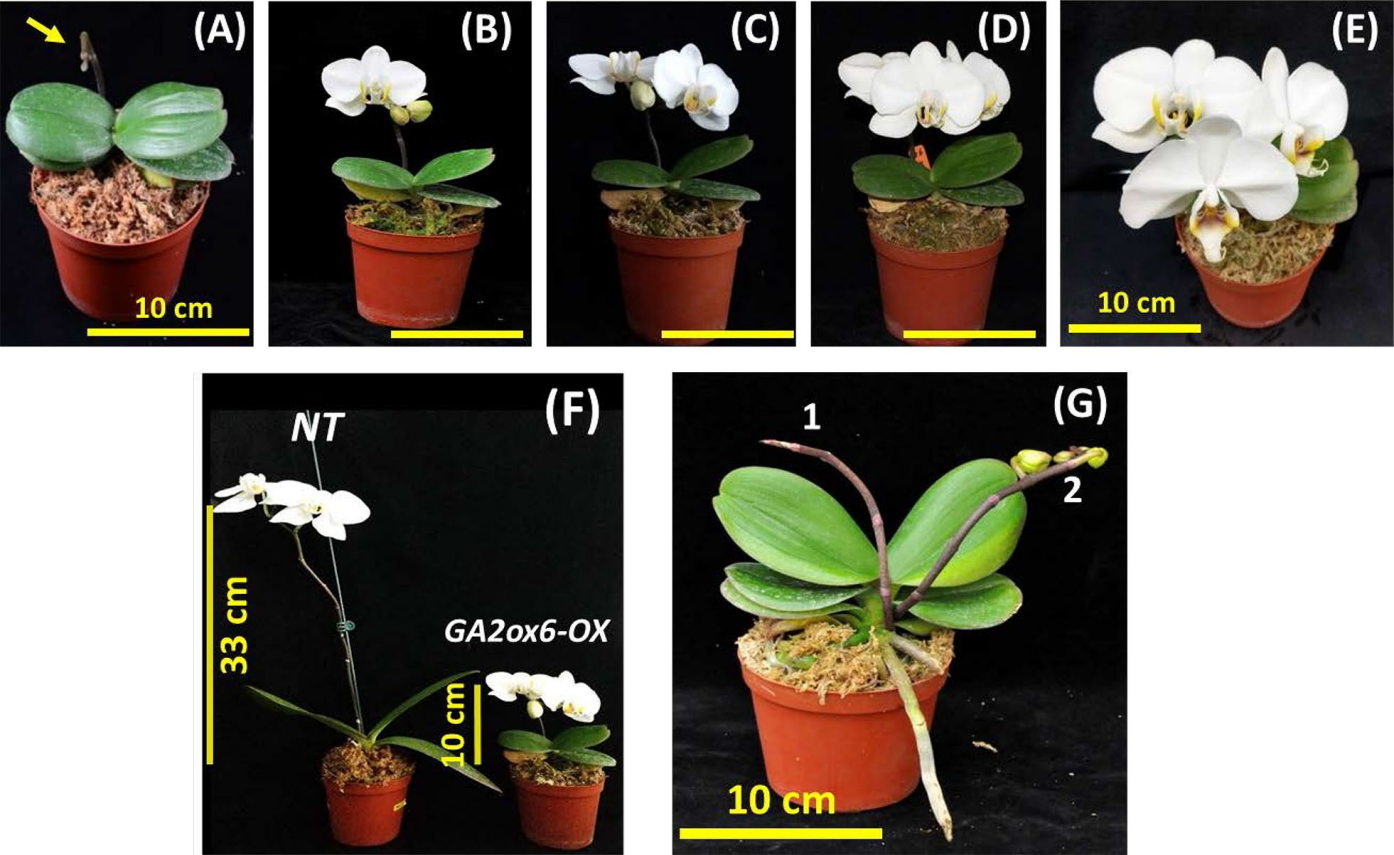
Phenotypic comparisons of *NT* and *GA2ox6*-*OX* transgenic plants at the blooming stage. **a** The *GA2ox6*-*OX* transgenic plants bear a flower spike (inflorescence) indicated by a yellow arrow. **b** Blooming of the first flower bud. Bar = 10 cm. **c** Blooming of the first two flower buds. Bar = 10 cm. **d** Blooming of all three flower buds. Bar = 10 cm. **e** Top view of completely blooming *GA2ox6*-*OX* transgenic plants. **f** Comparison of *NT* and *GA2ox6*-*OX* transgenic plants at the blooming stage. The 33 cm flower spike length of the *NT* line and 10 cm spike length of *GA2ox6*-*OX* plants are indicated by yellow lines. **g** An example of a *GA2ox6*-*OX* transgenic line with two flower spikes

The flower spikes of one *GA2ox6*-*OX* transgenic line took approximately two to three months to completely elongate, and then the first flower bud began to swell and bloom (Fig. 5a, b). A series of photographs shows the flowering process of one flower spike with 3 flower buds, from the blooming of the first flower to the end of the blooming of the third flower (Fig. 5b–e). In this case, each flower bud took approximately 10 days to complete blooming, and each flower lasted approximately 1 to 2 months for a total two- to three-month flowering period, similar to that of *NT* control plants.

Interestingly, the flower spike length (10 cm) of the *GA2ox6*-*OX* transgenic lines was much shorter than that (33 cm) of the *NT* line (Fig. 5f). Although the flower spike of the *GA2ox6*-*OX* transgenic lines was short, it still bore three flower buds with similar sizes and shapes and only one fewer bud than observed in the *NT* line (Fig. 5f). In addition, two *GA2ox6*-*OX* transgenic lines from more than 20 mature transgenic plants grew two flower spikes, while none were observed in *NT* control plants during the study period; whether these two flower spikes are related to the expression of *OsGA2ox6* requires further investigation. An example of the two flower spikes is shown in Fig. 5g.

## Discussion

### The C_20_-type *OsGA2ox6* does not affect the size of flower buds and is useful in miniaturizing Phalaenopsis orchids

There are two types of GA2oxs, distinguished by either the C_20_ or C_19_ type of GA substrate catalysis (Lo et al. 2008; Schomburg et al. 2003). The weaker reduction in plant size by C_20_-GA2ox than by C_19_-GA2ox might be attributed to substrate catalysis not being the limiting step in the GA biosynthesis pathway (Lo et al. 2008; Rieu et al. 2008) (Additional file 1: Fig S1). In addition, our previous studies demonstrated that overexpression of *OsGA2ox6* in rice and tobacco reduced plant size but had no significant effects on the reproductive organs (Lo et al. 2008). The present study showed that the expression of *OsGA2ox6* in *GA2ox6*-*OX* transgenic lines significantly reduced flower spike and leaf size but did not affect the size of flower buds (Figs. 3 and 5), thus making *OsGA2ox6* a useful gene for miniaturizing Phalaenopsis orchids.

### Miniaturization of Phalaenopsis orchids can be achieved by overexpressing the rice *OsGA2ox6* gene

Inhibitors of GA biosynthesis have been used as PGRs to reduce longitudinal shoot growth and for many other applications, such as miniaturizing plants or improving seedling acclimatization in ornamental horticulture (Latimer and Whipker 2013; Rademacher 1991). Among PGRs, PAC and UNI, two triazole-type compounds that retard plant growth by inhibiting GA synthesis, are frequently used in the miniaturization of Phalaenopsis orchids to reduce shipping costs and produce an aesthetically pleasing shape (Latimer and Whipker 2013; Newton and Runkle 2010; Rademacher 2000; Wang and Hsu 1994). However, repeated applications of PAC and UNI are needed to maintain the miniaturization of Phalaenopsis orchids, which raises the cost, and the effectiveness of this treatment can vary among plants in ways that significantly affect their market value.

To overcome these drawbacks, a transgenic approach involving the introduction of a gene involved in GA biosynthesis can be utilized. Many transformation methods that provide a feasible approach for the introgression of foreign genes to alter Phalaenopsis orchid phenotypes have been established (Anzai et al. 1996; Chai et al. 2002; Hsieh and Huang 1995; Hsing et al. 2016). In the present study, a rice C_20_-type GA2ox gene, *OsGA2ox6*, which affects active GA synthesis (Lo et al. 2008), was introduced and ectopically overexpressed in *Phalaenopsis* Sogo Yukidian ‘SPM313’ in order to miniaturize regenerated plantlets. Despite various transgenic events with different T-DNA copy insertions (Additional file 1: Fig S4), all the *GA2ox6*-*OX* transgenic lines showed the same phenotype (Fig. 3), and each individual line could be vegetatively propagated to maintain the same miniaturized phenotype observed in the maternal transgenic lines (data not shown). These observations suggest that Phalaenopsis orchids can be miniaturized by overexpressing the rice *OsGA2ox6* gene and that the miniaturized phenotype of these ectopically expressed transgenic lines can be uniformly propagated to ensure their market value. In addition, this transgenic approach using *OsGA2ox6* can be further expanded to different orchids or other flower species, which is more advantageous than using hybridization or mutation with *Phalaenopsis equestris*, as these methods are limited in *Phalaenopsis* genus.

### The low leaf L/W ratio and much shorter flower spike of *GA2ox6*-*OX* lines reduce the space needed for growth and handling

The *GA2ox6*-*OX* transgenic lines displayed darker-green, shorter and wider leaves (Fig. 3) than the *NT* lines, reflecting typical GA-deficiency phenotypes (Nir et al. 2014; Sakamoto et al. 2004; Varbanova et al. 2007). In addition to confirming this phenotype, the present study successfully demonstrated the recognition and expression of *OsGA2ox6* driven by the *ZmUbi* promoter and the functional effects of OsGA2ox6 in these Phalaenopsis orchids (Figs. 2, 3, 4).

The leaf shape, characterized by a low leaf L/W ratio, and the much shorter flower spike of these *GA2ox6*-*OX* transgenic lines (Figs. 3, 5) could reduce the space required for plantlets to grow in greenhouses and for packing boxes, which would improve the handling and transportation requirements of these orchids. In addition, the much shorter flower spike provides a novel flower pattern and does not require a plastic support stick (Fig. 5f); thus, these miniaturized Phalaenopsis orchids could provide a new style of orchid for tabletop decorations, attracting more consumers to orchids and expanding the market. In brief, these features that save costs would in many ways make these miniaturized Phalaenopsis orchids very competitive in the floral industry.

### The faster formation of PLBs in the *GA2ox6*-*OX* transgenic lines may be correlated with STM regulation and is beneficial for mass propagation

The in vitro clonal propagation of PLBs is a routine practice for mass production in the orchid floriculture industry (Yam and Arditti 2009). These PLBs (often described as somatic embryos) undergo a process of cell differentiation to form plantlets. During the regeneration and propagation process of the *GA2ox6*-*OX* transgenic lines, the PLBs of transgenic lines (Fig. 1m–o) propagated much faster and produced more PLBs, when wounded regions were more significant, than the *NT* line (Fig. 1l–n), resulting in less leaflet differentiation from PLBs. This phenomenon might be similar to the promotion of rice tillers under GA deficiency caused by the overexpression of *OsGA2ox* genes (Lo et al. 2008).

In *Arabidopsis*, the activity of shoot apical meristems was reported to be regulated by a class-I KNOTTED-like homeobox gene called SHOOT MERISTEMLESS (STM), which activated cytokinins and repressed GA biosynthesis (Jasinski et al. 2005). In addition, the process of cell differentiation in orchid tissue culture can be promoted by adding banana pulp since the banana pulp contains abundant natural cytokinins (Arditti and Ernst 1993; Withner 1974). Both cytokinins and GA are likely to be involved in the regulation of somatic embryogenesis (PLB formation) during orchid tissue culture. A recent study suggested that STM and GA2ox might also be involved in the regulation of PLB formation in *Phalaenopsis aphrodite*, as the expression pattern of *PaSTM* during PLB formation was positively correlated with that of *PaGA2ox1* and the overexpression of *PaSTM* in *Arabidopsis* accompanies the upregulation of *AtGA2ox2* (Fang et al. 2016). Our results showed that the overexpression of *OsGA2ox6* promoted the formation of large amounts of PLBs in the transgenic lines, reinforcing the potential for the expression of *GA2ox* genes to promote PLB formation via *PaSTM*. However, the mechanism underlying the promotion of PLB formation by GA2ox requires further investigation.

### Conclusion and future perspectives

In the present study, we demonstrated that the ectopic expression of *OsGA2ox6* can miniaturize *Phalaenopsis* Sogo Yukidian ‘SPM313’ without compromising its blooming ability, providing an alternative, useful method for miniaturizing Phalaenopsis species to produce a supply for the potential demands of the miniature Phalaenopsis orchid market.

GA2ox genes have been identified and characterized in many plant species (Lo et al. 2008; Schomburg et al. 2003; Schrager-Lavelle et al. 2019; Wuddineh et al. 2015), and various activities of individual *OsGA2ox* gene families (Hsieh et al. not yet published, in review) and different mutation variants of *OsGA2ox6* (Lo et al. 2017) have also been demonstrated. Therefore, the GA2ox genes from various plant species and variants of *OsGA2ox6* can be used to expand the toolbox for miniaturizing Phalaenopsis orchids. Although many GA2ox genes from different plant species have been studied, the GA2ox genes in Phalaenopsis have not yet been identified or functionally characterized. Research on orchids is now in the postgenomic era (Tsai et al. 2017), with transcriptomic databases such as *OrchidBase* (Tsai et al. 2013) and *Orchidstra* (Chao et al. 2017) that contain numerous gene-expressed sequences collected from Phalaenopsis species. These genes provide an excellent resource for functional studies of GA2ox genes in Phalaenopsis species, and GA2ox genes with known functions can be further applied to miniaturize Phalaenopsis orchids.

## Supplementary information

**Additional file 1: Fig S1.** A simplified GA biosynthesis and metabolic pathway. The target gene (KO) inhibited by PGRs, such as PAC and UNI, is indicated. The GA2 oxidases that catalyze the C_20_ and C_19_ types of GA substrates in their relative pathways are shown. **Fig S2.** Propagation of PLBs and plantlet regeneration. When the PLBs grew to approximately 2-4 mm (A), their tip portions were cut horizontally (B) to expose their epidermal/surface cells (C) in order to form/propagate new PLBs and generate plantlets (D). **Fig S3.** GUS staining results for PLBs and root tips of *NT* and *GA2ox6-OX* transgenic lines. (A) GUS staining of tissues from *NT* and *GA2ox6-OX* lines are shown. (B) GUS staining of PLBs from *NT* and *GA2ox6-OX* lines are shown. (C) GUS staining of root tips from *NT* and *GA2ox6-OX* lines are shown. **Fig S4.** Southern blot analysis. Genomic DNA isolated from leaves of the *NT* line and three *OsGA2ox6* transgenic lines (#1-1, #3-2 and #3-3) was cut with the restriction enzyme *HindIII* and probed with α P^32^-labeled GUS DNA fragment. **Fig S5.** Phenotypic comparisons of *NT* and *GA2ox6-OX* transgenic lines. Three plants each from the *NT* line and four *GA2ox6-OX* transgenic lines (#1-1, #3-2, #3-3, and #3-4) were compared. The growth stages of plants from line #1-1 are earlier than those of the *NT* line and the other 3 *GA2ox6-OX* lines. All *GA2ox6-OX* lines showed similar phenotypes, but their phenotypes differed from those of the *NT* line. The scale bar = 10 cm.

**Additional file 2: Table S1.** Primers and their sequences used in this study.

## Abbreviations

AS: Acetosyringone
GA2ox6-OX: *OsGA2ox6* overexpression
NT: Nontransgenic
PAC: Paclobutrazol
PGRs: Plant growth retardants
PLBs: Protocorm-like bodies
UNI: Uniconazole
