# Supplementary figures and images for: Phalaenopsis orchid miniaturization by overexpression of OsGA2ox6, a rice GA2-oxidase gene
Source: Bot Stud. 2020 Apr 6;61:10. doi: 10.1186/s40529-020-00288-0 (PMC7136379; doi:10.1186/s40529-020-00288-0)

Fig S1

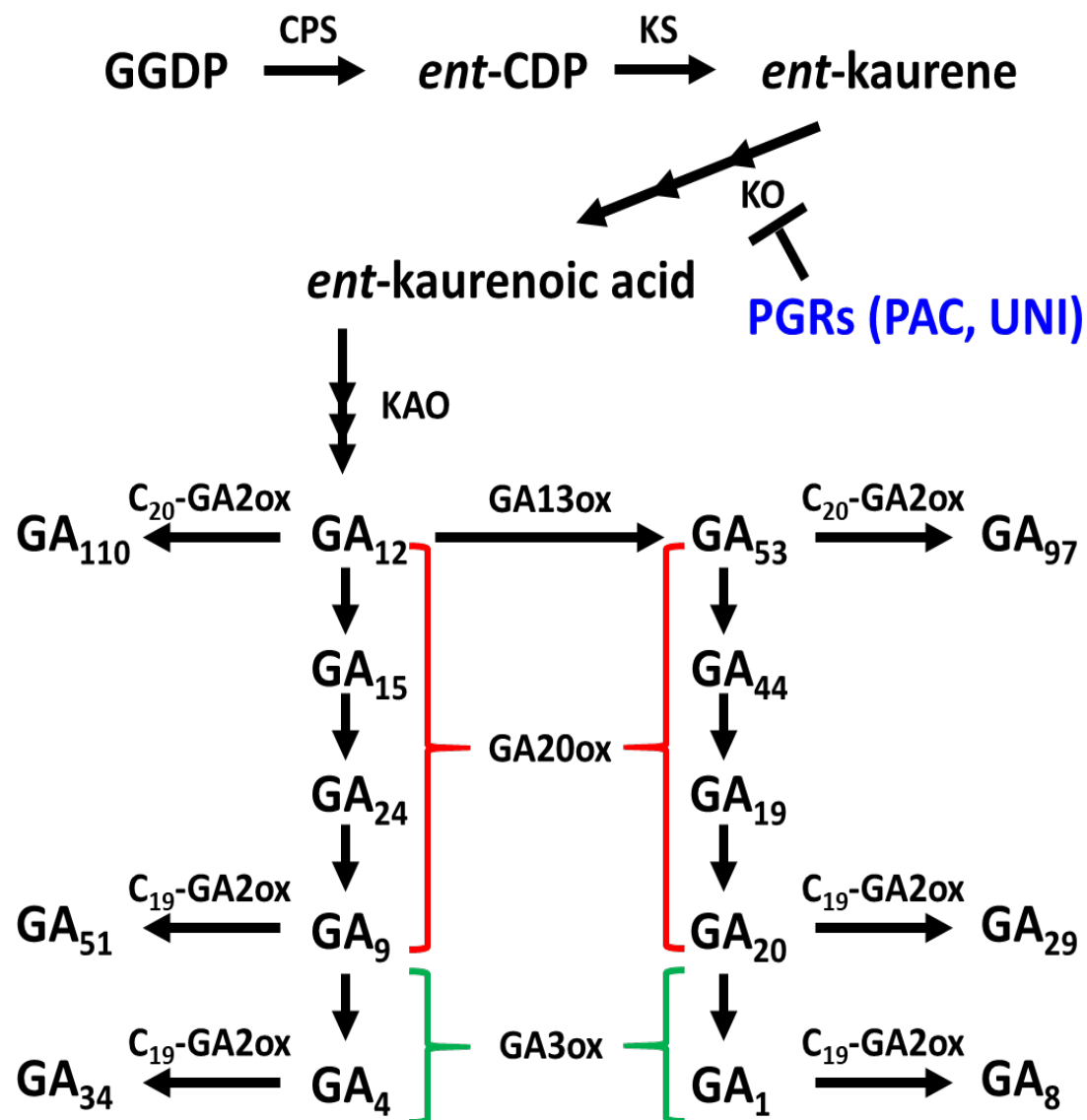

**Fig S2**

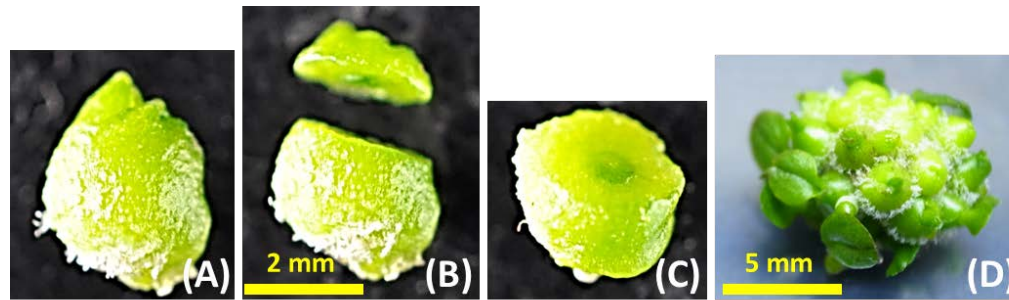

**(A) GUS staining with tissues in tubes**

**Fig S3**

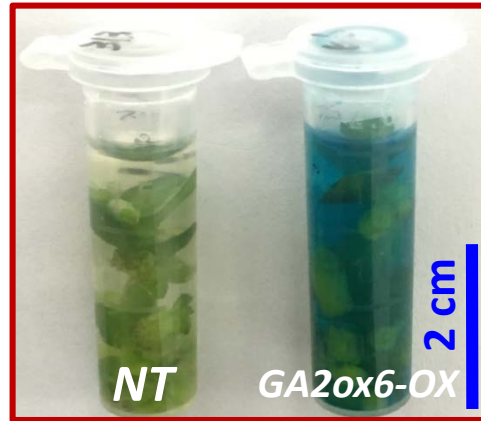

**(B) PLBs**

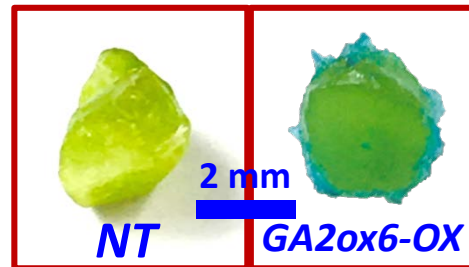

**(C) Root tips**

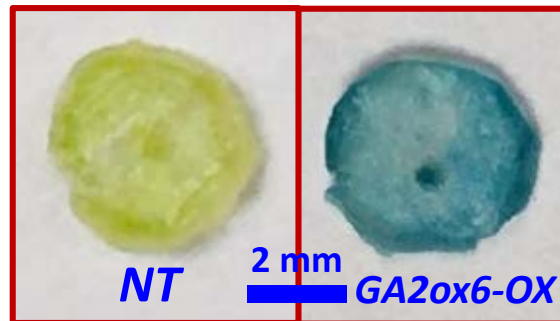

**Fig S4**

**Southern blot**

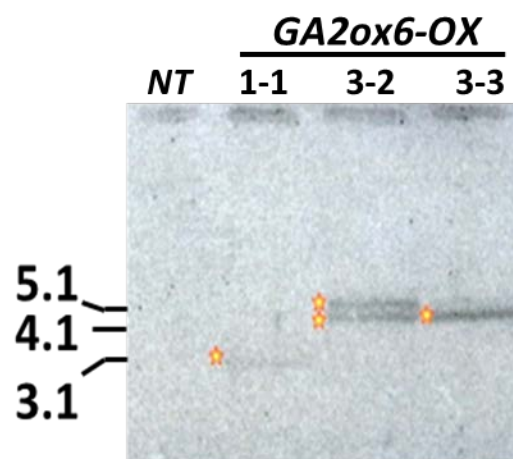

Fig S5

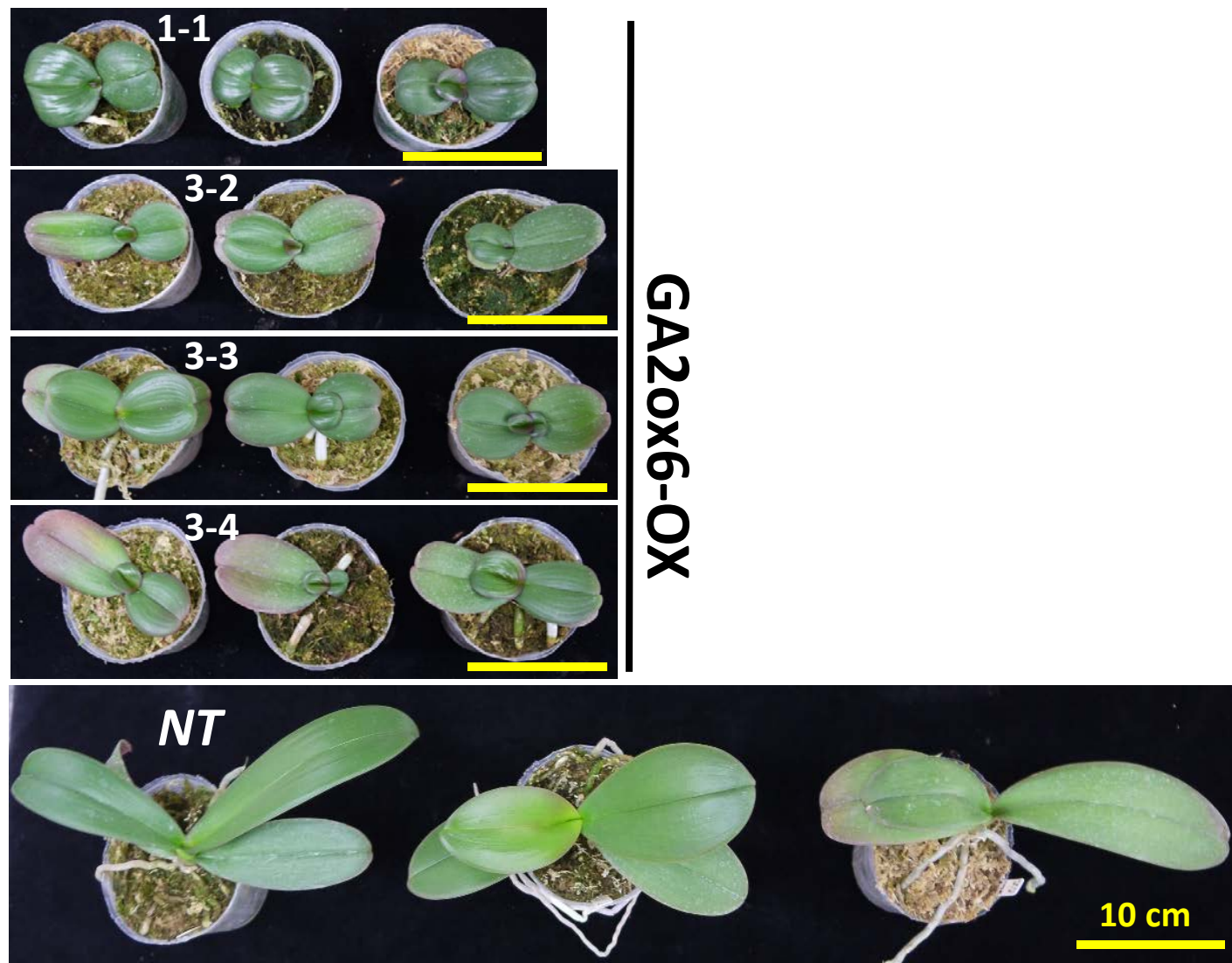

Supplement: Supplementary file 1 — Additional file 1: Fig S1. A simplified GA biosynthesis and metabolic pathway. The target gene (KO) inhibited by PGRs, such as PAC and UNI, is indicated. The GA2 oxidases that catalyze the C20 and C19 types of GA substrates in their relative pathways are shown. Fig S2. Propagation of PLBs and plantlet regeneration. When the PLBs grew to approximately 2-4 mm (A), their tip portions were cut horizontally (B) to expose their epidermal/surface cells (C) in order to form/propagate new PLBs and generate plantlets (D). Fig S3. GUS staining results for PLBs and root tips of NT and GA2ox6-OX transgenic lines. (A) GUS staining of tissues from NT and GA2ox6-OX lines are shown. (B) GUS staining of PLBs from NT and GA2ox6-OX lines are shown. (C) GUS staining of root tips from NT and GA2ox6-OX lines are shown. Fig S4. Southern blot analysis. Genomic DNA isolated from leaves of the NT line and three OsGA2ox6 transgenic lines (#1-1, #3-2 and #3-3) was cut with the restriction enzyme HindIII and probed with α P32-labeled GUS DNA fragment. Fig S5. Phenotypic comparisons of NT and GA2ox6-OX transgenic lines. Three plants each from the NT line and four GA2ox6-OX transgenic lines (#1-1, #3-2, #3-3, and #3-4) were compared. The growth stages of plants from line #1-1 are earlier than those of the NT line and the other 3 GA2ox6-OX lines. All GA2ox6-OX lines showed similar phenotypes, but their phenotypes differed from those of the NT line. The scale bar = 10 cm. [file 40529_2020_288_MOESM1_ESM.pdf]

Table S1. Primers and their sequences used in this study


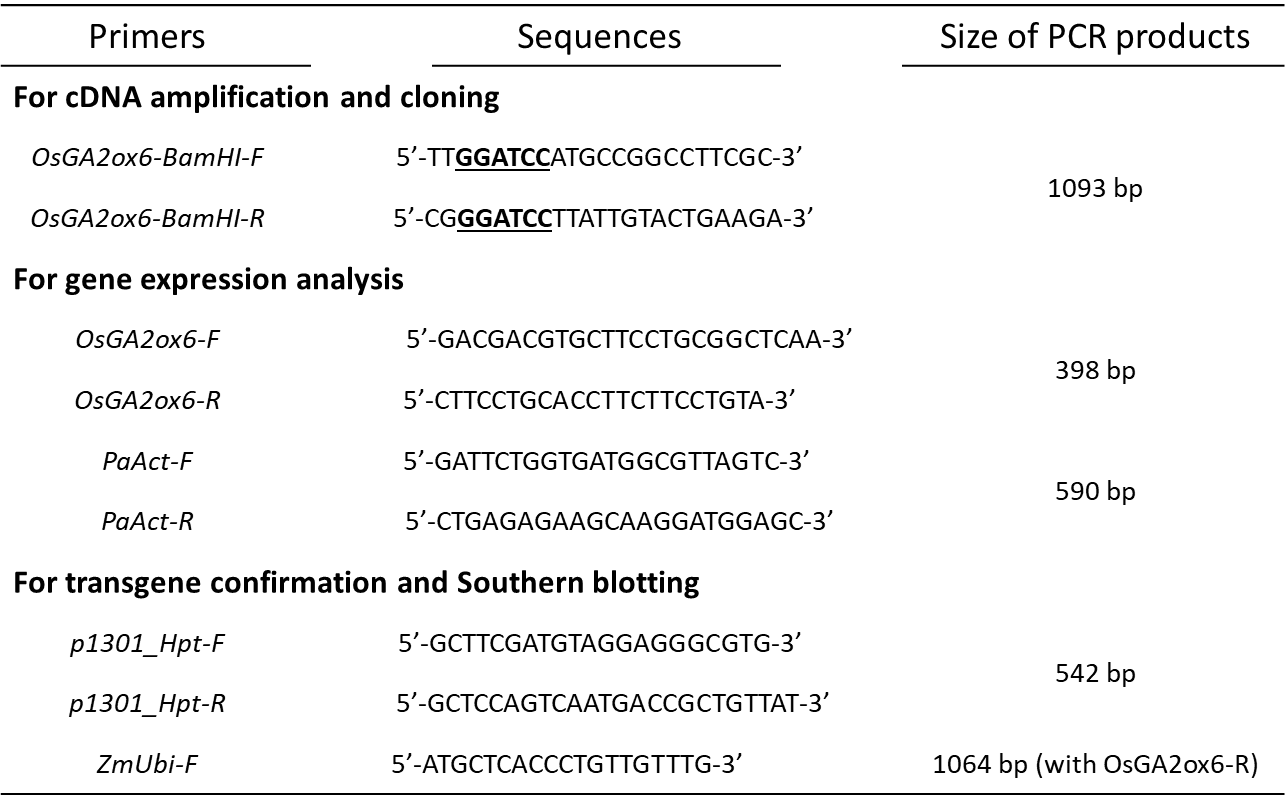

Supplement: Supplementary file 2 — Additional file 2: Table S1. Primers and their sequences used in this study. [file 40529_2020_288_MOESM2_ESM.docx]
